# Current status of *Helicobacter pylori* infection and prevalence of resistance-associated gene mutations in Bortala Mongolian Autonomous Prefecture, Xinjiang: a single-center study

**DOI:** 10.3389/fmicb.2025.1641773

**Published:** 2025-10-16

**Authors:** Gai-Ling Yuan, Jing Yang, Yan Peng, Jin-Xin Lai, Zheng-Kang Li

**Affiliations:** ^1^The Fifth Division Hospital of Xinjiang Production and Construction Corps, Bole, China; ^2^Laboratory Medicine, Guangdong Provincial People’s Hospital (Guangdong Academy of Medical Sciences), Southern Medical University, Guangzhou, China

**Keywords:** *Helicobacter pylori*, resistance genes, capsule sampling, Bortala Mongolian Autonomous, antimicrobial resistance patterns

## Abstract

**Background:**

*Helicobacter pylori* (*H. pylori*), a globally prevalent infectious pathogen, has been epidemiologically associated with dyspepsia, peptic ulcer disease, and gastric carcinogenesis. However, comprehensive regional epidemiological data regarding infection prevalence, antimicrobial resistance patterns, and associated risk factors remain insufficient in the Bortala MongolianAutonomous Prefecture of Xinjiang, China.

**Methods:**

A cross-sectional study was conducted from June 4 to June 9, 2023, enrolling 341 participants through systematic sampling in Bortala Prefecture. Diagnostic procedures combined non-invasive urea capsule sampling with genotypic detection techniques for simultaneous *H. pylori* identification and antimicrobial resistance profiling. Multivariate logistic regression analysis was performed on demographic and behavioral variables obtained through standardized questionnaires.

**Results:**

The study revealed an *H. pylori* infection prevalence of 29.03% (99/341; 95% CI: 24.13–34.34%). Genotypic resistance analysis demonstrated resistance rates of 51.52% (51/99; 95% CI: 41.72–61.32%) for clarithromycin and 46.46% (46/99; 95% CI: 36.66–56.26%) for levofloxacin. Female participants showed a marginally elevated infection risk (OR = 1.27, *p* = 0.35), while communal cup-sharing behavior demonstrated borderline significance as a risk factor (OR = 1.95, *p* = 0.09). No statistically significant independent risk factors were identified in the multivariate analysis.

**Conclusion:**

The Bortala Prefecture exhibits alarming genotypic resistance patterns to first-line antibiotics, despite a moderate *H. pylori* infection prevalence. These findings underscore the critical need for region-specific antimicrobial stewardship and evidence-based treatment protocols guided by local resistance profiles. Future research should prioritize stratified sampling methodologies to delineate ethnicity-specific risk profiles and validate potential risk factors in larger cohorts.

## Introduction

In a groundbreaking study conducted in 1983, Barry J. Marshall and J. Robin Warren successfully isolated and characterized the bacterium *Helicobacter pylori* (*H. pylori*) within the gastric mucosa through innovative biopsy culture techniques (Marshall and Warren, 1984). This discovery revolutionized the field of medical microbiology by challenging the long-held belief in gastric sterility that had dominated gastroenterology for decades. Through meticulous experimentation involving both animal models and human clinical trials, Marshall and colleagues established a clear causal link between *H. pylori* colonization and various gastroduodenal pathologies, demonstrating through double-blind therapeutic trials that targeted antimicrobial therapy could lead to complete disease resolution. Their rigorous methodology included electron microscopic visualization of spiral bacteria in inflamed gastric tissue and successful fulfillment of Koch’s postulates through experimental reinfection studies (Malfertheiner et al., 2014). These findings provided irrefutable epidemiological and molecular evidence that gastric diseases previously attributed to stress or dietary factors could instead be directly linked to *H. pylori* infection. In recognition of their paradigm-shifting work, Warren and Marshall were awarded the Nobel Prize in Physiology or Medicine in 2005, with the Nobel Committee particularly noting their “tenacity and willingness to challenge prevailing dogmas” (Pincock, 2005).

*Helicobacter pylori* is a Gram-negative, microaerophilic bacterium with a unique ability to persistently colonize the human gastric environment through multiple evolutionary adaptations. This pathogen has evolved intricate mechanisms to survive the harsh acidic conditions of the stomach, including chemotactic navigation through the mucus layer and selective adhesion to gastric epithelial cells, facilitating long-term infections and subsequent pathological outcomes (Salama et al., 2013). One key biochemical adaptation is the production of urease, an enzyme that catalyzes urea hydrolysis to generate ammonia, effectively neutralizing stomach acid and creating a localized pH-neutral microenvironment (Ansari and Yamaoka, 2017). The bacterium’s spiral morphology and polar flagella further enhance its motility through viscous gastric mucus. Additionally, *H. pylori* employs a sophisticated arsenal of virulence factors including cytotoxin-associated gene A (CagA), vacuolating cytotoxin A (VacA), and outer membrane proteins that target host cell signaling pathways, modulating inflammatory responses and causing diverse injuries ranging from epithelial erosion to malignant transformation (Fu and Lai, 2022). Clinically, *H. pylori* infection demonstrates a spectrum of outcomes from asymptomatic colonization to life-threatening conditions, being definitively linked through population-based cohort studies to chronic gastritis (95% association), peptic ulcers (70–80% of cases), gastric adenocarcinoma (1–3% lifetime risk), and MALT lymphoma (Burucoa and Axon, 2017). The International Agency for Research on Cancer (IARC) has classified *H. pylori* as a Group 1 carcinogen based on consistent oncogenic pathway activation observed across diverse populations (Cancer IAFRO, 1994). Modern eradication regimens combining proton pump inhibitors with clarithromycin-based triple therapy have demonstrated 85–90% success rates, with longitudinal studies showing that successful *H. pylori* elimination reduces gastric cancer risk by 35–45%, contributing to declining incidence rates in regions implementing widespread screening programs (Lee et al., 2016).

The epidemiology of *H. pylori* infection exhibits significant geographical heterogeneity, influenced by regional characteristics, public health infrastructure, and socioeconomic development indices (Hooi et al., 2017). In a multicenter retrospective cohort study, significant antimicrobial resistance patterns were observed in urban Chinese adults diagnosed with *H. pylori* infections, with clarithromycin resistance at 50.83% and levofloxacin resistance at 47.17% (Wang et al., 2024). These findings highlight concerning trends in antibiotic resistance profiles among *H. pylori*-infected populations, particularly regarding gender-based susceptibility differences.

Bortala Mongolian Autonomous Prefecture in Xinjiang is a multi-ethnic settlement area, with diverse health habits and dietary cultures among its inhabitants, including Mongolians, Han Chinese, Kazaks, and other ethnic groups. The traditional Mongolian diet, rich in fermented dairy products, may modulate the gastric microbiota and inhibit *H. pylori* colonization. Conversely, the custom of sharing utensils may increase the risk of cross-infection. Comprehensive regional epidemiological data regarding infection prevalence, antimicrobial resistance patterns, and associated risk factors remain insufficient in this region. This study aims to comprehensively assess the epidemiological characteristics of *H. pylori* infection, including prevalence rates, genotypic resistance patterns, and associated risk factors, in this region.

## Materials and methods

### Study design

A cross-sectional study was conducted in the Bortala region of Xinjiang from June 4 to June 9, 2023, to investigate the prevalence of *H. pylori* infection. The survey was promoted online and targeted individuals aged 18 to 60 years. Exclusion criteria included: (1) Individuals who had taken antibiotics, bismuth agents, antimicrobial traditional Chinese medicine, or proton pump inhibitors and H2 receptor antagonists within the past month. (2) Individuals who had received *H. pylori* eradication treatment within the past three months. (3) Pregnant or breastfeeding women. (4) Individuals with severe cardiac, hepatic, or renal insufficiency, severe neurological disorders, or mental illnesses. This study adhered to ethical principles and national regulations, including the Declaration of Helsinki. All participants provided informed consent. Participants were enrolled randomly and voluntarily, without any specific incentives. Trained physicians were present to guide and assist participants in filling out the survey questionnaires. The study was approved by the Ethics Committee of the Guangdong Provincial People’s Hospital (Ethics approval number: KY-Q-2022-384-02).

### Questionnaire survey

The questionnaire, developed by Professor Gu Bing’s team from the Guangdong Provincial People’s Hospital, covered the following aspects:1. Demographic Characteristics: Gender, age stratification (<30 years, 30–39 years, 40–49 years, and 50–60 years), anthropometric measurements (height and weight), marital status (single or married), and educational attainment (elementary education or below, secondary education including high school or vocational training, and tertiary education)0.2. Lifestyle Assessment: Dietary patterns, tobacco use, alcohol intake, tea consumption behaviors, and communal dining utensil sharing practices. Body mass index (BMI) was calculated using the standard formula: weight in kilograms divided by height in meters squared (kg/m^2^). According to the World Health Organization (WHO) classification, participants were categorized as underweight (BMI < 18.5 kg/m^2^), normal weight (BMI 18.5–24.9 kg/m^2^), overweight (BMI 25.0–29.9 kg/m^2^), and obese (BMI ≥ 30.0 kg/m^2^).

### *Helicobacter pylori* infection status

For gastric juice collection, we used a minimally invasive string test with a commercial disposable sampler kit (Hongmei Diagnostic Technology Co., Ltd., Shenzhen). Participants drank water and swallowed a capsule with a sampling string attached, with one end of the string secured to the cheek. After 60 min, the capsule dissolved, and the gastric juice was absorbed by the sampling string, which was then retrieved from the stomach. The sampling string was cut at the indicated position using sterile scissors and transferred to a preservation solution (Tris/saline/EDTA). All samples were sent to the Guangdong Clinical Gene Testing Quality Control Center of the Guangdong Provincial People’s Hospital for subsequent qPCR-based genetic analysis (Wang et al., 2023. The flowchart of the string test gastric fluid sample collection procedure).

Nucleic acid extraction was performed using the Stream SP96 automated nucleic acid extractor (DaAn Gene Co., Ltd., China) and a commercial genomic DNA extraction kit (DaAn Gene Co., Ltd.). Real-time fluorescent quantitative PCR amplification was carried out using the Gentier 96R real-time PCR system (TianLong Technology Co., Ltd.). The detection targeted the urease A gene specific to *H. pylori* (*H. pylori* nucleic acid detection kit, Hongmei Diagnostic Technology Co., Ltd., Shenzhen). The amplification protocol used was as follows: 2 min at 42 °C, 2 min at 95 °C, followed by 40 cycles, each consisting of 10 s at 95 °C and 45 s at 58 °C. A sample was considered positive for *H. pylori* infection if the cycle threshold (Ct) value was below 35 and showed a typical S-shaped curve. For *H. pylori*-positive samples, resistance to clarithromycin (A2142G, A2143G, and A2142C in the 23S rRNA gene) and levofloxacin (A260T, C261A, T261G, G271A, G271T, and A272G in the *gyrA* gene) was detected using the *H. pylori* 23S rRNA and *gyrA* mutation detection kit (Hongmei Diagnostic Technology Co., Ltd., Shenzhen). A Ct value below 30 with a typical S-shaped curve was considered indicative of resistance.

### Statistical analysis

We used the chi-square test to analyze the relationship between *H. pylori* infection and variables such as gender, age, BMI, education level, and marital status. The sample size was calculated based on the observed infection rate (29.03%), a 5% error margin, and a 95% confidence level. Ninety-five percent confidence intervals were calculated for all statistical indicators, including infection rate and antibiotic resistance rate. Factors with *p* values less than 0.5 in the univariate logistic regression analysis, as well as factors previously found to be significantly associated with *H. pylori* infection in prior studies (such as gender, age, education level, and marital status), were included in the multivariate logistic regression analysis to further assess their relationship with *H. pylori* infection. Independent risk factors were identified and confirmed through univariate and multivariate logistic regression analyses, with odds ratios (OR) and 95% confidence intervals calculated to reflect their impact (*p* < 0.05). All analyses were performed using SPSS 27.0 software.

## Results

### Basic information of participants

A total of 341 subjects were enrolled in this research for gastric fluid specimen collection. The mean age of the cohort was 44.68 years, comprising 34.02% (*n* = 116) male participants and 65.98% (*n* = 225) female participants. Within the study population, 65.4% exhibited BMI measurements falling within the standard range of 18.5–25, while 27.3% demonstrated BMI values between 25–30. Over half of the cohort attained advanced education levels, with 59.5% possessing an advanced academic qualification. The study documented 271 married participants, representing 79.5% of the total cohort (Table 1. Characteristics of the Study Population).

**Table 1:** Characteristics of the study population.

Characteristic	Total (*n* = 341)	Infection (*n* = 99)	Uninfected (*n* = 242)	Infection rate (%)	*p* value
Gender					
Male	116	30	86	25.86	0.04
Female	225	69	156	30.67	
Age (years)					
<30	50	17	33	34.00	0.67
30–39	60	17	43	28.33	
40–49	70	20	50	28.57	
50–60	161	45	116	27.95	
BMI (Kg/m^2^)					
Underweight	3	1	2	33.33	0.94
Normal weight	222	62	160	27.93	
Overweight	94	29	65	30.85	
Obese	22	7	15	31.82	
Education level					
Lower secondary education and below	61	15	46	24.59	0.07
Intermediate: high school or vocational training	77	20	57	25.97	
Higher education	203	64	139	31.52	
Marital Status					
Unmarried	70	22	48	31.43	0.23
Married	271	77	194	28.41	

### *Helicobacter pylori* infection rate and antibiotic resistance in the urban population of Bortala region, Xinjiang

The survey conducted among the urban population of the Bortala region in Xinjiang revealed a *Helicobacter pylori* infection prevalence of 29.03% (99/341 cases, 95% confidence interval: 24.28–33.78%). A Genotypic resistance analysis demonstrated substantial resistance patterns among infected subjects, with clarithromycin resistance observed in 51.52% of isolates (51/99, 95% CI: 41.60–61.44%) and levofloxacin resistance detected in 46.46% of cases (46/99, 95% CI: 36.66–56.26%). Notably, dual resistance to both antibiotics was identified in 32.32% of the resistant strains. The findings highlight not only the considerable burden of *H. pylori* infection in this northwestern Chinese population but also reveal alarmingly high resistance rates exceeding those reported in many coastal urban centers. (Table 2. Survey of *H. pylori* Infection Rate and Resistance Rate in the Urban Population of Bortala Region, Xinjiang).

**Table 2:** Survey of *H. pylori* infection rate and resistance rate in the urban population of Bortala Region, Xinjiang.

Detection target	Total, *n*	Positive, *n*	Positive rate (%)	95% CI (%)
*H. pylori*	341	99	29.03	24.13–34.34
Clarithromycin	99	51	51.52	41.72–61.32
Levofloxacin	99	46	46.46	36.66–56.26

### Risk factors associated with *Helicobacter pylori* infection in the Bortala region of Xinjiang

Table 3 univariate and multivariate logistic regression analysis of *H. pylori* infection.

**Table 3:** Univariate and multivariate logistic regression analysis of *H. pylori* infection.

Variables	Cases/Total (%)	Univariate logistic regression analysis		Multivariate logistic regression analysis	
		OR (95% CI)	*p* value	OR (95% CI)	*p* value
Gender					
Male	30/116 (25.86)	1.0 (Reference)		1.0 (Reference)	
Female	69/225 (30.67)	1.27 (0.77–2.1)	0.35	1.15 (0.68–1.95)	0.60
Age (years)					
<30	17/50 (34.00)	1.0 (Reference)		1.0 (Reference)	
30–39	17/60 (28.33)	0.77 (0.34–1.73)	0.53	0.75 (0.32–1.76)	0.51
40–49	20/70 (28.57)	0.78 (0.36–1.69)	0.53	0.76 (0.34–1.71)	0.51
50–60	45/161 (27.95)	0.75 (0.38–1.47)	0.40	0.73 (0.36–1.47)	0.38
Education level					
Higher education	64/203 (31.52)	1.0 (Reference)		1.0 (Reference)	
Primary school or lower	15/61 (24.59)	0.71 (0.37–1.37)	0.31	0.69 (0.35–1.37)	0.29
Intermediate: high school or vocational training	20/77 (25.97)	0.77 (0.44–1.36)	0.37	0.75 (0.42–1.35)	0.34
Marital Status					
Unmarried	22/70 (31.43)	1.0 (Reference)		1.0 (Reference)	
Married	77/271 (28.41)	0.87 (0.49–1.55)	0.64	0.85 (0.47–1.54)	0.59
BMI (Kg/m^2^)					
Normal weight	63/223 (28.25)	1.0 (Reference)		1.0 (Reference)	
Underweight	1/3 (33.33)	1.27 (0.11–14.8)	0.85	1.3 (0.11–15.2)	0.83
Overweight	28/93 (30.11)	1.09 (0.64–1.87)	0.75	1.07 (0.62–1.86)	0.80
Obese	7/22 (31.82)	1.18 (0.47–2.99)	0.73	1.15 (0.44–2.97)	0.78
Separate dining					
No	70/229 (30.57)	1.0 (Reference)		1.0 (Reference)	
Yes	29/112 (25.89)	0.80 (0.48–1.34)	0.4	0.82 (0.48–1.41)	0.47
Drinking tea					
No	68/239 (28.45)	1.0 (Reference)		1.0 (Reference)	
Yes	31/102 (30.39)	1.09 (0.66–1.81)	0.74	1.06 (0.63–1.80)	0.82
Drinking alcohol					
No	75/252 (29.76)	1.0 (Reference)		1.0 (Reference)	
Yes	24/89 (26.97)	0.88 (0.51–1.52)	0.64	0.85 (0.48–1.50)	0.57
Sharing water cup					
No	86/311 (27.65)	1.0 (Reference)		1.0 (Reference)	
Yes	13/30 (43.33)	2.0 (0.93–4.28)	0.08	1.95 (0.89–4.25)	0.09
Smoking					
No	88/304 (28.95)	1.0 (Reference)		1.0 (Reference)	
Yes	11/37 (29.73)	1.04 (0.49–2.20)	0.92	1.02 (0.47–2.20)	0.97

This table presents the comprehensive analysis of risk factors associated with *H. pylori* infection within the Bortala cohort population. The gender-based analysis revealed comparable infection rates between males (25.86%, 30/116) and females (30.67%, 69/225), with no statistically significant disparity observed (*p* = 0.35). Age stratification demonstrated that the youngest demographic (<30 years) exhibited the highest prevalence at 34.00% (17/50), though statistical testingconfirmed no significant variation across age groups.

Body mass index analysis showed elevated infection rates in both obese (31.82%, 7/22) and underweight individuals (33.33%,1/3) compared to normal weight subjects (28.25%, 63/223). However, logistic regression models adjusting for potential confounders revealed non-significant associations (obese: OR 1.18, 95% CI 0.47–2.99, *p* = 0.73; underweight: OR 1.27, 95% CI 0.11–14.80, *p* = 0.85). Educational attainment analysis demonstrated a graduated pattern, with the lowest infection rate in the low education group (junior high school or below: 24.59%, 15/61) compared to secondary education (25.97%, 20/77) and higher education groups (31.52%, 64/203). The protective trends remained non-significant after adjustment (secondary education: OR 0.77, 95% CI 0.44–1.36; low education: OR 0.71, 95% CI 0.37–1.37).

Marital status analysis showed married individuals had a modestly reduced infection rate (28.41%, 77/271) versus unmarried counterparts (31.43%, 22/70), though not statistically significant (OR 0.87, 95% CI 0.49–1.55). Lifestyle factor investigation revealed intriguing patterns: adherents of separate dining practices showed 25.89% infection (29/112) versus 30.57% in shared dining groups (70/229), while tea drinkers exhibited 30.39% prevalence (31/102) versus 28.45% non-tea drinkers (68/239). Alcohol consumers demonstrated 26.97% infection (24/89) compared to 29.76% abstainers (75/252). Smoking status analysis showed comparable rates between smokers (29.73%, 11/37) and non-smokers (28.95%, 88/304). None reached statistical significance in univariate analysis.

Multivariate logistic regression incorporating gender, age categories, education levels, and marital status failed to identify any significant independent predictors (all *p* > 0.15). Notably, shared drinking cup practice showed borderline significance across both univariate (OR 2.00, 95% CI 0.93–4.28) and multivariate models (OR 1.95, 95% CI 0.89–4.29), with *p*-values approaching 0.08–0.09 threshold. This potential association warrants verification in larger cohort studies with enhanced statistical power.

## Discussion

This study was designed to investigate the epidemiological characteristics of *H. pylori* infection, genotypic resistance patterns, and associated risk factors within the urban population of Bortala Mongol Autonomous Prefecture, Xinjiang Uyghur Autonomous Region, China. The implementation of non-invasive diagnostic modalities for detecting *H. pylori* infection and genotypic resistance patterns represents a critical advancement in clinical microbiology. A seminal study by Losurdo et al. validated the clinical efficacy of stool-based molecular assays for precise identification of *H. pylori* colonization and resistance profiles (Losurdo et al., 2020). However, given the potential confounding effects of complex fecal microbiota composition, our investigation employed a minimally invasive string test coupled with quantitative *p*olymerase chain reaction (qPCR) analysis of gastric fluid specimens. This innovative sampling methodology effectively mitigates the invasiveness associated with endoscopic biopsy procedures while obtaining representative gastric luminal samples. Furthermore, gastric fluid analysis circumvents sampling bias inherent in the focal distribution patterns of *H. pylori* colonization within gastric mucosal niches (Yang et al., 2023). The qPCR platform enables multiplex detection of bacterial genomic targets through strategic selection of species-specific primers and antibiotic resistance-associated probes. This methodological integration facilitates concurrent multi-target detection through strategic primer and probe selection, permitting simultaneous assessment of *H. pylori* infection status and clarithromycin/levofloxacin resistance determinants.

The observed infection rate in our study exceeds the 27.08% prevalence reported in Chinese urban populations using identical detection protocols. However, comparative analysis reveals significantly lower rates relative to epidemiological data from other Chinese cities, particularly Quanzhou where infection rates were documented at 52.60% (Xie et al., 2025). This discrepancy may be attributable to the inclusion of multi-ethnic populations in prior multicenter studies, wherein all documented ethnic groups exhibited higher *H. pylori* infection rates compared to the Han ethnicity (Peng et al., 2023). Notably, our study cohort comprised 90% Han participants. Future research should prioritize implementing stratified sampling methodologies to delineate ethnicity-specific risk profiles. Additionally, the genotypic resistance patterns observed in our study highlight alarming rates of clarithromycin and levofloxacin resistance, exceeding 15% (Zeng et al., 2024). These findings underscore the urgent need for region-specific antimicrobial stewardship and evidence-based treatment protocols guided by local resistance profiles.

Although the present investigation examined multiple risk factors associated with *H. pylori* infection, the exclusion of socioeconomic status and familial predisposition may introduce residual confounding. Epidemiological evidence from prior studies consistently demonstrates that these omitted variables constitute established risk factors for *H. pylori* transmission (Zhou et al., 2023). Subsequent research should therefore incorporate these covariates to enhance the comprehensiveness of risk stratification models. Notably, the current analysis did not identify statistically significant independent predictors of *H. pylori* infection through multivariable regression. This null finding may be attributable to methodological limitations inherent in observational studies, population-specific characteristics, or unmeasured confounders, necessitating validation through prospective cohort studies with enhanced covariate ascertainment.

This study’s primary strength resides in the implementation of high-precision detection methodologies for *H. pylori* identification. However, this investigation exhibits several methodological constraints that warrant consideration. Firstly, the cross-sectional design inherently precludes the establishment of causal relationships between *H. pylori* infection and potential risk factors. Secondly, non-stratified random recruitment may compromise population representativeness, particularly given the demographic composition of the Bortala cohort. While the study population encompasses multiple ethnic groups, the predominant Han majority and limited sample size restrict extrapolation of findings to broader populations. Future research should prioritize stratified sampling methodologies to enhance populationrepresentativeness.

In conclusion, our study provides valuable epidemiological insights into *H. pylori* infection dynamics and resistance patterns in the Bortala region. The findings highlight the need for targeted infection control programs and optimized antimicrobial stewardship strategies to address the high prevalence of antibiotic resistance. Future research should focus on larger, stratified cohorts to validate these findings and explore the underlying mechanisms of resistance and transmission.

## Conclusion

In summary, our investigation of *H. pylori* infection among urban residents in Bortala Mongolian Autonomous Prefecture, Xinjiang Uygur Autonomous Region, China, revealed a prevalence of 29.03% and alarming genotypic resistance patterns, with 51.52% of clinical isolates exhibiting clarithromycin resistance and 46.46% displaying levofloxacin resistance. These findings underscore the critical need for region-specific antimicrobial stewardship and evidence-based treatment protocols guided by local resistance profiles. Future research should prioritize stratified sampling methodologies to delineate ethnicity-specific risk profiles and validate potential risk factors in larger cohorts. The high prevalence of antibiotic resistance identified in this study necessitates urgent exploration of alternative therapeutic strategies, particularly bacteriophage-antibiotic combination regimens that may exert synergistic bactericidal effects against multidrug-resistant strains (Haq et al., 2024). This investigation provides valuable epidemiological insights and forms an evidence base for developing targeted infection control programs to disrupt *H. pylori* transmission pathways in Bortala.

## Data Availability

The original contributions presented in the study are included in the article/supplementary material, further inquiries can be directed to the corresponding authors.
